# Implementation of Micro-EDM Monitoring System to Fabricate Antimicrobial Nanosilver Colloid

**DOI:** 10.3390/mi13050790

**Published:** 2022-05-18

**Authors:** Kuo-Hsiung Tseng, Meng-Yun Chung, Juei-Long Chiu

**Affiliations:** 1 Department of Electrical Engineering, National Taipei University of Technology, Taipei 10608, Taiwan; f10473@mail.ntut.edu.tw; 2 Business Planning Development Department, Panasonic Eco Solution Sales Taiwan Co., Ltd., Taipei 10608, Taiwan; chiu.paul@tw.panasonic.com

**Keywords:** electrical discharge machine, energy consumption, nanosilver colloid, antimicrobial

## Abstract

This study implemented a discharge energy and success-rate monitoring system to replace the traditional oscillograph observation method and conducted a microbial control test for a nanosilver colloid prepared by an Electrical Discharge Machine (EDM). The advantage of this system is that the discharge conditions can be instantly and continuously observed, and the optimized discharge parameter settings can be recorded. The monitoring system can use the arcing rate to control the energy consumption of the electrodes to standardize the nanosilver colloid. The results show that the arcing rate, electrode weight loss, and absorption peak wavelength are very accurate. The nanosilver colloid prepared by EDM is free of any chemical additive, and in comparison to other preparation methods, it is more applicable to biotechnology, even to the human body. The microbial control test for the nanosilver colloid included a Bathroom sample, Penicillium, Aspergillus niger, and Aspergillus flavus. In test solution NO.1 (prepared by micro-EDM), the effects of all four samples were inhibited at 14mm in a metal ring experiment, and in the cotton pad experiment, Penicillium was inhibited at 17 mm. In the metal ring experiment, test solution NO. 2 (prepared by EDM) had an effect at 20 mm on the bathroom samples, but at only 15 mm on flavus. In the cotton pad experiment, the inhibited effect was more effective in Penicillium and Aspergillus Niger; both inhibited effects occurred at 25 mm. Test solutions NO.3 (prepared by micro-EDM) and NO.4 (32 ppm Ag+) had a 14–15 mm effect on all samples in the metal ring experiment. In the cotton pad experiment, NO.3 had an effect on Penicillium at 19 mm while the effect on the others occurred at 14 mm, and NO.4 had an effect at 25 mm in Penicillium and Aspergillus Niger, and only at 14 mm in the bathroom and Aspergillus flavus samples.

## 1. Introduction

Nanotechnology, biotechnology, and information technology are three basic technologies in the 21st century [1,2]. In Germany in 1984, H. Gleiter developed the first nanoparticle, initiating the application of nanotechnology. Scientists refer to powder particle aggregates in material particles with a diameter smaller than 100 nm as nanoparticles. When silver is made at a nanoscale, the particles are minified while the surface is enlarged so that the functions of the silver are greatly increased. With the appearance of new functions, many new applications of nanosilver have been developed [3]. Nanotechnology discusses new physical and chemical characteristics of technology at a scale under 100 nm. In recent years, the development of nanotechnology and nanomaterial has gradually entered the application phase. In terms of the current conditions and trends of the development of the nanosilver market, according to a Future Markets investigation, the global nanosilver application market accounted for USD 17.8 billion in 2011. In total, 95% of nanosilver market applications are based on the antibacterial property of nanosilver; the majority of those applications are in the cosmetic and medical markets, with an output value of about USD 8.4 billion, accounting for 47% of the nanosilver application market. The market applications of nanosilver in textiles and the packing industry account for 17% and 8% of nanosilver market applications, respectively. Other nanosilver market applications, mostly for high-level electronic materials, account for 5%. Since the effect of nanomaterial on the human body is yet uncertain, nanosilver has been conservatively applied in the cosmetic and medical markets. Consumers’ concerns regarding the safety of nanoproducts influence the market output value of nanosilver in cosmetics, medical treatments, textiles, and packing [4,5,6].

In addition, as the nanosilver technique gradually becomes mature, the application of nanosilver to the market of paints, pigments, and coating is estimated to increase in the future [7,8]. In terms of the biomedical industry, in order to guarantee the advantage of talent in biotechnology competition in the 21st century, the Ministry of Education added six original, major resource centers in the rising field of “biomedical nano, stem cell, and tissue engineering” in 2004, becoming the instructional resource centers for seven major fields. The general objective is to cultivate top biotechnical talents with foresight; cross-domain, enhanced industrial experience; and an international view. According to statistics from the Industrial Development Bureau, Ministry of Economic Affairs, the nanomaterials commonly used in Taiwan are TiO_2_, SiO_2_, and nanosilver, and the annual input of labor, financial resources, material resources, and turnover has increased in recent years. Nanotechnology development has been an important technical means for adding industrial value [9]. The main goals of this study are the following: (1) to implement a real-time monitoring system (discharge energy and success rate) for a micro-Electrical Discharge Machine (micro-EDM) with instant and continuous observation of the results, and to record the optimized discharge parameter settings; (2) to use ESDM to fabricate an environmental-friendly nanosilver colloid to inhibit widespread fungi that are harmful to the human body, including those from a bathroom sample, Penicillium, Aspergillus niger, and Aspergillus flavus.

## 2. Materials and Methods

### 2.1. Preparation of Nanosilver Colloid by Electrical Spark Discharge Method (ESDM)

There are two ways to fabricate nanoparticles: one is physical, and the other is a chemical method. The chemical way uses the “bottom-up” method, which is similar to the “self-assembly” technology of substances and functional substances produced by nature. These methods are all constructed from the level of the molecule to the nanoscale. The physical preparation method is generally a “top-down” etching technique. Mechanical pulverization and grinding use higher-hardness materials as a medium to grind particles by shear force, friction force, and impact force to break down the particles to a small size.

The electrical discharge machine (EDM) is traditionally used for processing metal materials with high toughness and hardness [10,11]. Its principle is that, when the switch is turned on, a spark is generated to consume the metallic contact surface. However, contact will become loose if the consumption occurs over time [12,13,14]. Therefore, the EDM takes advantage of the principle to put the processing electrode and the object in an insulation liquid [15]. When the two electrodes are placed at a very small distance (μm), the electrons will flow from the cathode to the anode and damage the insulation state of the insulation liquid. The electrons continuously impact on the surface of the object [16]. This will generate a high spark with a high temperature. The spark temperature is around 6000 °C to 10,000 °C, and it will melt the surface into micro- or nanoparticles [17,18]. Using the principle of an EDM to fabricate the nanoparticle is called the Electrical Spark Discharge Method (ESDM).

The proposed EDM system mainly consists of the following sub-systems:

1. Power system: 100 DC volts and above;
2. Servo control system: controls the *z*-axis motor to maintain the two electrodes at a distance of micrometers;
3. Parameter control panel: adjusts the discharge cycle or *z*-axis speed, etc.

**Figure 1:**
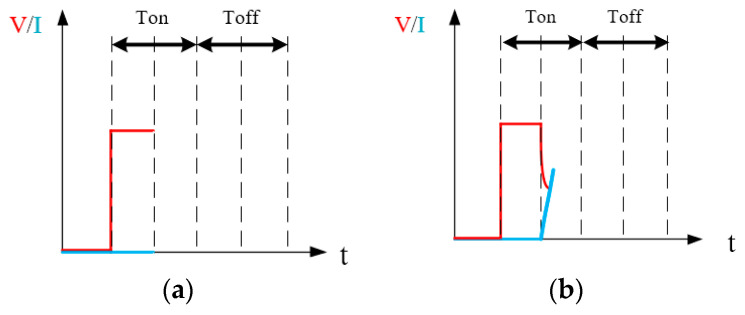

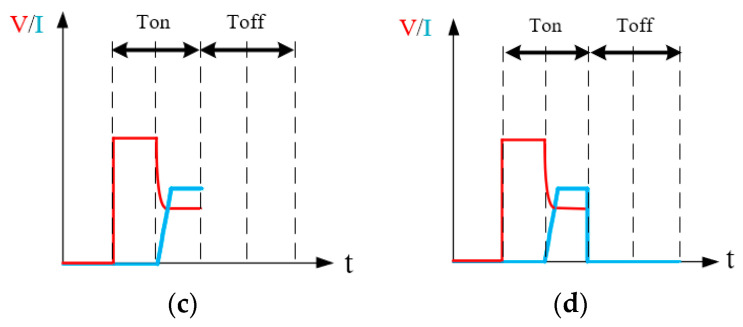
Electrical Spark Discharge Method (discharge success): (**a**) voltage without current; (**b**) discharge start; (**c**) spark discharge occurs; (**d**) discharge off.

The EDM monitoring system has traditionally used an oscillator to observe the voltage and current wavelengths between electrodes, as shown in Figure 1 [19,20,21]. T_on_ is the discharging time, when the electrodes will successfully carry out spark discharge; T_off_ is the stopping time. The period (T_on_ + T_off_) will continuously repeat during the processing time, for example, 20 min and 40 min in this study. When the discharge is successful, the period T_on_ when the pulse voltage appears is composed of a period of spark discharge ignition delay time and a period of spark discharge time. When the discharge is successful, the initial stage of the pulse voltage is the ignition delay time, and the electric field strength provided by the pulse voltage during this period is not enough to break down the dielectric of the electrode gap. Therefore, the voltage between the gap (V_IEG_) will be maintained at the open circuit voltage, and the current between the gap (I_IEG_) will be 0A, as shown in Figure 1a. The dielectric strength of the electrode gap gradually decreases during the ignition delay of the spark discharge. The period after the dielectric in the electrode gap is broken down by the electric field strength is the spark discharge period. During the spark discharge, the electrode gap presents a low-resistance state, so I_IEG_ rapidly rises to a maximum value, and V_IEG_ drops to a very low voltage value, during which spark discharge occurs between the tool and the workpiece, as shown in Figure 1b. During a successful spark discharge time, the spark will be generated, as shown in Figure 1c. When the power pulse voltage is turned off during T_off_, the electrode gap will immediately end the spark discharge state because no pulse voltage provides the energy required for discharge. During the T_off_ period, I_IEG_ and V_IEG_ both drop to zero, as shown in Figure 1d. During T_off_, the electrode gap will gradually recover the insulation, and the insulation degree of the electrode gap at the end of T_off_ will affect the ignition delay time of the next cycle of discharge. In addition, after a successful electrode discharge, the deionized water (DW) will be restored to a state of insulation in order to facilitate the next cycle of discharge and eliminate the metallic particles between the electrodes. Therefore, the T_on_-T_off_ settings affect processing efficiency and quality [22]. There are three scenarios of discharge [23,24,25] as shown in the following:

1. Discharge failure (situation 1): There is voltage without a current. This is the same as with an open circuit.
2. Discharge failure (situation 2): As the two electrodes are very close, it is likely to cause a short circuit, similar to a current without voltage.
3. Discharge success: The spark damages the DW insulation and successfully generates a spark discharge. In addition, there are voltage and a current at the same time.

Nanometal colloids, such as nanogold, nanosilver, nano-copper, nano-aluminum, and nano-titanium, can be made with any method able to conduct electricity using an EDM [26,27,28,29,30]. Nanosilver colloids have been applied in biomedical research, and silver ions can resist bacteria growth. For example, an enzyme with silver ion cannot ferment [31,32].

The nanometal colloids can be analyzed using a spectrophotometer, which can measure two indicative parameters:

1. Absorption peak: The instrument will emit UV and visible light to radiate the object under analysis. Through the sensor receiving the light, it can measure the absorption peak of each wavelength of the colloid. Higher absorption peaks suggest higher concentrations of colloid at the wavelength. On the contrary, a lower absorption rate suggests a lower concentration of colloid [33,34,35].
2. Wavelength of the highest absorption peak: Nanoparticles will radiate under the stimulation of certain wavelengths. For example, nanosilver has the highest absorption value around 400 nm [36], while nano-copper is around 280 nm [37].

As an EDM during discharge has no cyclic changes, and the frequency is very high, the discharge feature cannot be observed using an oscillator [38]; however, it can be observed by capturing an instant image of it. The disadvantage of the traditional method is that it cannot accumulate the successful discharge times. In other words, using an oscillator to observe the efficiency of the EDM is very inconvenient. This study proposed a monitoring system that can realize real-time discharge monitoring and obtain the statistics for successful discharge times, electrode energy consumption, and discharge success rate.

**Figure 2:**
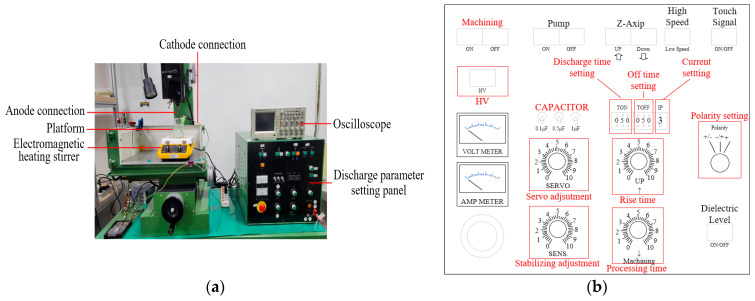
(**a**) Picture of EDM. (**b**) Discharge parameter setting panel.

The EDM vaporizes the silver wire into nano-sized silver particles. The working electrodes and workpiece in this study are distinguished as electrodes. The electrode connected to positive electricity is the anode, while that connected to negative electricity is the cathode. The nanosilver colloid is prepared by ESDM in this study. The silver wire (with a diameter of 1 mm) is ground into nano-sized (1~100 nm) silver nanoparticles by the high temperature of the ESDM [39]. The discharge parameter setting panel is used to adjust the process parameters of the EDM, and the setting panel provides a voltmeter and ammeter for measuring the mean values of the voltage and current between the electrodes. Figure 2a is a diagram of the EDM and its discharge parameter setting panel. The anode and cathode connection connects the two electrodes in the beaker on the platform. The electromagnetic heating stirrer is used to stir the deionized water inside the beaker. The reason is that the electrode gap will gradually recover the insulation, and the insulation degree of the electrode gap at the end of T_off_ will affect the ignition delay time of the next cycle of discharge, so the stirring moves the nanoparticles away from the gap between the electrodes to make the gap recover the insulation more quickly. The oscilloscope is used to observe the voltage and current of the gap between the electrodes. In Figure 2b, Z-Axis is used to control the rise/fall of the *Z*-axis. CAPACITOR is used to set the capacitance value and is proportional to the transient current of discharge pulse at T_on_. T_on_ and T_off_ control the discharge and off time of the electrodes. Polarity adjusts the polarity of the upper and lower electrodes. SERVO controls the servo motor speed and is proportional to the motor speed. Stabilizing controls the sensitivity of the feedback circuit and is also proportional to the motor speed. HV switches the DC power supply from 140 V to 240 V. In addition, Ip is proportional to the discharge current and processing rate, and inversely proportional to precision.

**Figure 3:**
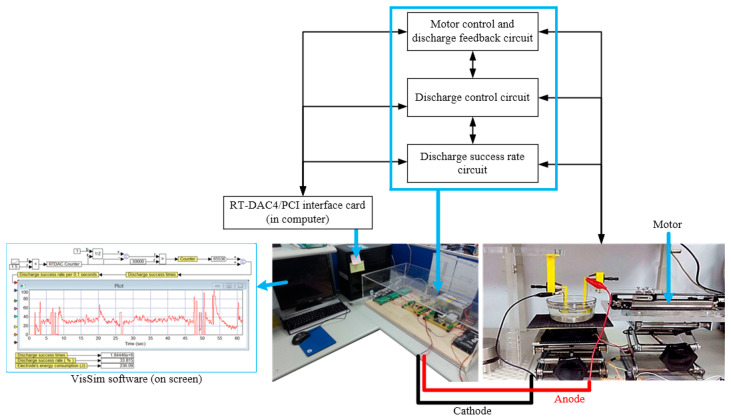
Picture of micro-EDM.

This study integrated multi-field technology research and successfully developed a micro-EDM set as shown in Figure 3. This micro-EDM was composed of an EDM jig mechanism, a hardware circuit system, and a software monitoring interface. In terms of design, the application of 3D printing and PLA materials can greatly reduce the manufacturing cost and the size of the equipment. The 3D-printed fix jig can hold the electrodes in the beaker. The hardware circuit part is designed from the circuit design of a PCB board combined with electronic components to achieve a large electric discharge machine. The PCB boards are the following: 1. motor control circuit and discharge feedback circuit (for controlling the motor to move forward and backward according to the feedback of the voltage); 2. discharge control circuit (for giving the output of the 100V DC pulse wave at the electrodes); and 3. discharge success rate circuit (for calculating the discharge success times, rate, and energy consumption with the software) [40,41,42]. The electrical spark discharge function and signal feedback are realized by VisSim software 6.0 (Visual Solution Inc., Pleasant Prairie, the USA) and an RT-DAC4/PCI interface card. Through this interface card, the software can be used to replace local electronic circuits to achieve the goals of a smaller circuit size and lower cost. Unlike the EDM, which needs an oscilloscope to observe the voltage and current of the gap between the electrodes and is not likely to count the discharge success times, the micro-EDM can accomplish these jobs with VisSim software. Because the micro-EDM is self-made, any repair is clear and easy. Although both an EDM and a micro-EDM can fabricate a nanosilver colloid by ESDM, the micro-EDM can make smaller-sized particles and better suspension colloids than the EDM does.

### 2.2. Activation and Cultivation of Strains

#### 2.2.1. Inhibition Zone Test

**Figure 4:**
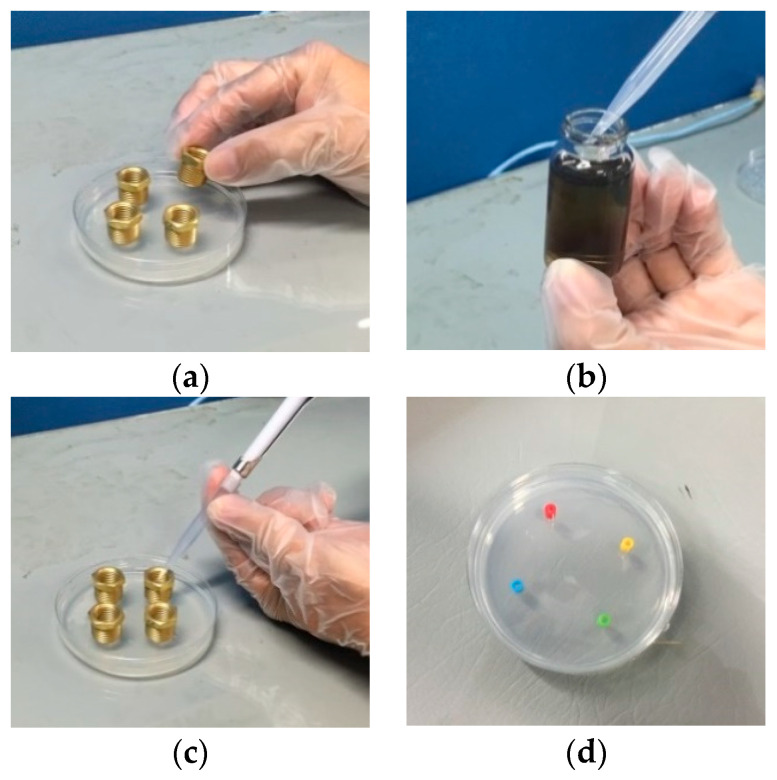
Pictures of inhibition zone test process: (**a**) metal rings placed on culture medium; (**b**) pipette extracts 0.15 mL test solution; (**c**) NO.1~NO.4 test solutions injected into metal rings, respectively; (**d**) metal rings carefully removed 30 min later and signs placed.

The other main purpose of the study was to use the traditional EDM and the micro-EDM that produced the nanosilver colloid to evaluate its effectiveness in the microbial control of fungi; as the particle size, zeta potential, and concentration were different, so its physical properties and chemical properties were also different. The study procedures were divided into following:

1. Test solution preparation: NO.1 was the nanosilver colloid made using the micro-EDM, with a silver ion concentration of 5 ppm. NO.2 was the nanosilver colloid produced by the traditional EDM; the silver ion concentration was 32 ppm, and the silver particle concentration was about 300 ppm. NO.3 was the nanosilver colloid produced by the micro-EDM; while the silver ion concentration was 5 ppm, the nanoparticle was the smallest. NO.4 was a 32 ppm silver ion titer.
2. Fungus sample preparation: There are four kinds of fungus samples: (a) the strain was randomly collected from the dim and damp parts of a bathroom; the strain was smeared on the Potato Dextrose Agar (PDA) and cultured; (b) penicillium: the strain was collected from moldy oranges; the strain was smeared on PDA and cultured; (c) Aspergillus Niger: pure strain was bought from BCRC (Bioresource Collection and Research Center, Hsinchu, Taiwan); the strain was smeared on Malt Extract Agar (MEA) and cultured; (d) Aspergillus Flavus: pure strain was bought from BCRC; the strain was smeared on PDA and cultured. The bathroom sample, Penicillium, Aspergillus niger, and Aspergillus flavus are the four targets in this study. These are widespread both indoors and outdoors, and those exposed to them will sometimes have allergies, get sick, or suffer an infection or other conditions that may harm the body. These four targets are easily obtained, simple to cultivate, and most importantly, they are often sources of trouble in normal life in Taiwan (due to the wet weather). Therefore, this study used the micro-EDM to fabricate a nanosilver colloid that can inhibit the four targets [43,44].
3. Experimental method design: Inhibition zone test: the size of the inhibition zones formed by the NO.1~NO.4 test solutions in four fungus culture media were observed in order to judge the microbial control effect of each test solution. The test process is shown in Figure 4. The test configuration is shown in Table 1. The NO.1~NO.4 test solutions were injected into the inhibition zones in the culture media cultivating different strains, respectively. The corresponding color labels are: NO.1 (Red), NO.2 (Yellow), NO.3 (Green), NO.4 (Blue).

#### 2.2.2. Microbial Control Cotton Pad Test

**Figure 5:**
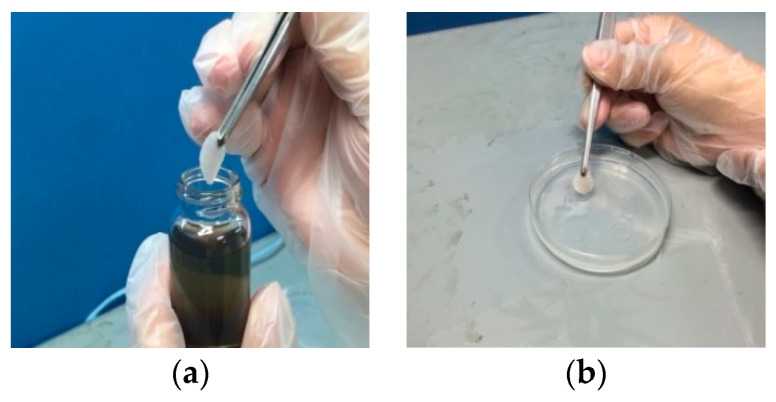

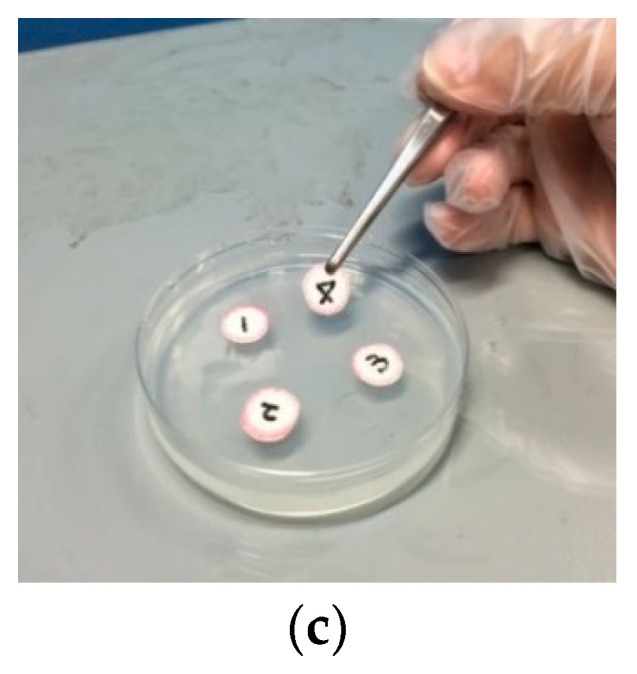
Pictures of cotton pad test process: (**a**) 140 mm labeled cotton pad soaked in NO.1~NO.4 test solutions; (**b**) cotton pad carefully placed in culture medium with tweezers; (**c**) the aforesaid steps repeated four times.

Cotton pads with a diameter of 140 mm were labeled 1, 2, 3, and 4, soaked in the NO.1~NO.4 test solutions, respectively, and placed in the culture media cultivating different strains; the inhibition zone sizes of the cotton pads were observed to judge the microbial control effects [45]. The test process is shown in Figure 5. The corresponding symbols are: NO.1 (1), NO.2 (2), NO.3 (3), NO.4 (4).

## 3. Results

### 3.1. The Results of the Nanosilver Colloid

**Figure 6:**
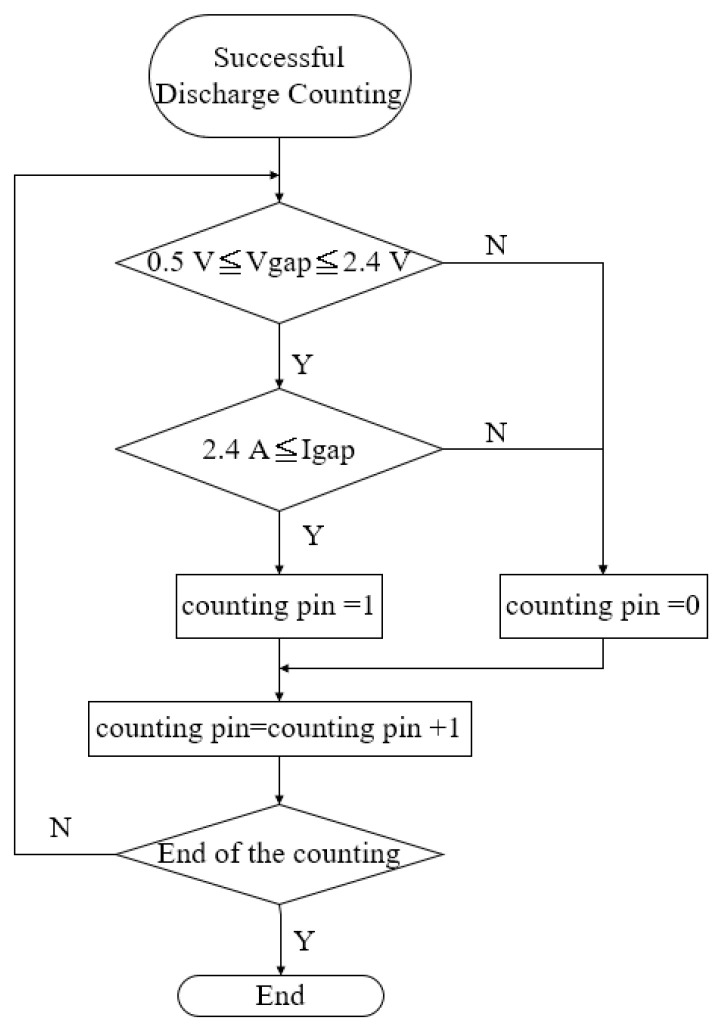
Flow chart of discharge success rate counting.

**Figure 7:**
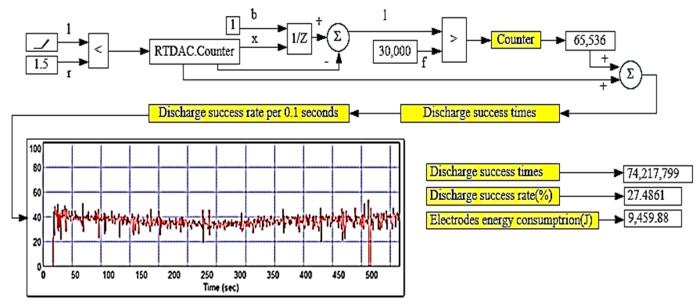
VisSim monitoring of the wave pattern.

**Figure 8:**
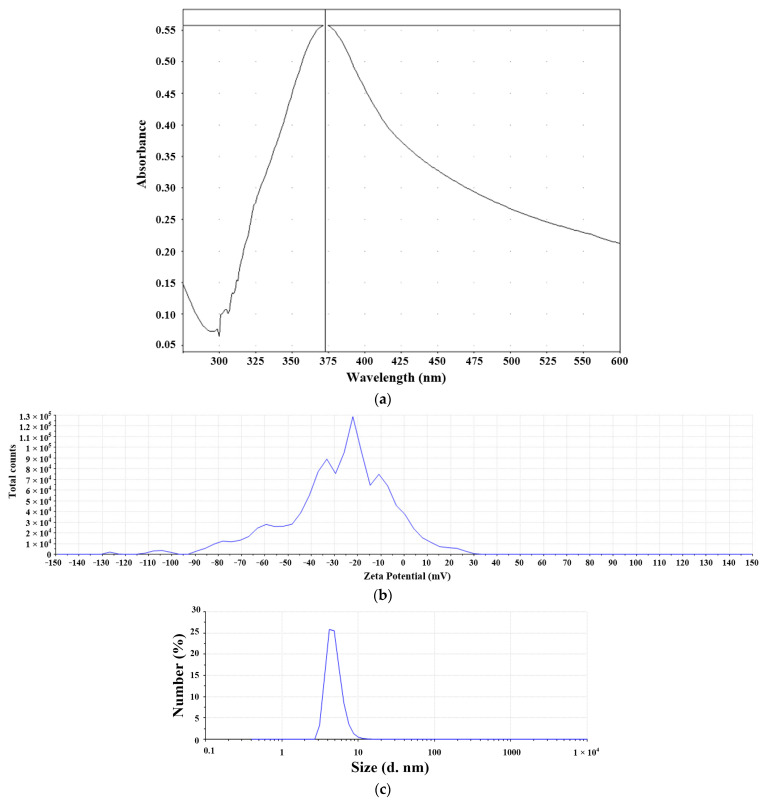
Characteristics of nanosilver colloid prepared using micro-EDM: (**a**) UV absorption wavelength; (**b**) zeta potential; (**c**) number PSD.

The micro-EDM was used to prepare the nanosilver colloid for test solution NO.1, and the related parameter settings are described in Table 2 and Table 3. The weight loss of the experiment was only 0.38 mg, which means only 0.38 mg nanoparticles were in the DW, as shown in Table 4. In addition, the silver ion concentration was about 5 ppm. In the discharge success rate circuit, the voltage signals must first be processed using two series resistors to divide the interelectrode gap voltage V_gap_. The V_gap_ was 0.0526 times that of the interelectrode gap voltage. The I_gap_ signals of the circuit were equal to the current that flows through the electrode gap. The conditions for the circuit to judge the successful discharge were 0.5 V ≦ V_gap_ ≦ 2.4 V and I_gap_ ≥ 2.4 A. If the discharge was successful (met the criteria above), the signal output from the circuit to the counting pin of the RT-DAC4/PCI card of the VisSim was 1 (counting pin = 1); on the contrary, if the discharge was not successful, the counting pin = 0. The discharge success rate was monitored through the signal transmission output by the discharge success rate circuit to the CNT0 counter of the RT-DAC4/PCI card, and the signal was processed by VisSim to obtain the discharge success rate and the cumulative number of successful discharges (summing all the counting pins during the processing time). The flow chart of the discharge success rate counting is shown in Figure 6. The calculation of discharge success rate is shown in Equations (1) and (2), where N_D_ is the cumulative number of successful sampling and discharging events in time T_on_; N_total_ is the total number of discharge samplings in time T_on_; D_cycle_ is the duty cycle (T_on_ + T_off_); t_proc_ is the production process time; and f_sample_ is the sampling frequency. This study was able to obtain the total discharge success times and determine the signals through this digital input/output interface card. It can replace the complex electrical circuit, which can count more than 1 million times per minute. The VisSim software used in this study is PC-based. By using the PC-based VisSim, this study was able to observe the real-time discharge times. In addition, it immediately computed the successful discharge times, electrode energy consumption, and discharge success rate, as shown in Figure 7. The success rate was 27.4861%, and the energy consumption was 236.09J. With these settings, this study was able to obtain the nanosilver characteristics. The UV wavelength of the nanosilver was 398 nm, and the absorbance was 0.56, as shown in Figure 8a. The zeta potential was −28.2 mV, as shown in Figure 8b. The size was around 5~8 nm, as shown in Figure 8c.

(1) $$\mathrm{Discharge}\;\mathrm{success}\;\mathrm{rate}=\frac{N_{D}}{N_{\mathrm{total}}}$$

(2) $$N_{\mathrm{total}}=D_{\mathrm{cycle}}\times t_{\mathrm{proc}}\times f_{\mathrm{sample}}$$

**Figure 9:**
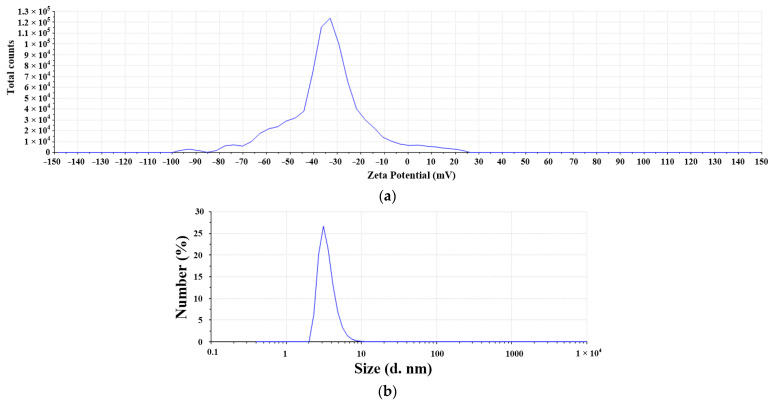
Characteristics of nanosilver colloid prepared using EDM: (**a**) zeta potential distribution; (**b**) number PSD.

The traditional EDM was used to prepare the nanosilver colloid for test solution NO.2, and the related parameter settings are described in Table 5 and Table 6. The weight loss of the experiment was only 124.43 mg, which means only 124.43 mg nanoparticles were in the DW, as shown in Table 7. The prepared nanosilver colloid was kept still for 2~3 weeks and then filtered through filter paper, which removed about 77.28 mg of precipitates and big silver particles; the estimated concentration was (124.43–77.28)/0.25 = 308 ppm. The UV wavelength of the nanosilver was 398 nm, and the absorbance was 1.1. The zeta potential was −33 mV, as shown in Figure 9a. The size was around 5~8 nm, as shown in Figure 9b.

**Figure 10:**
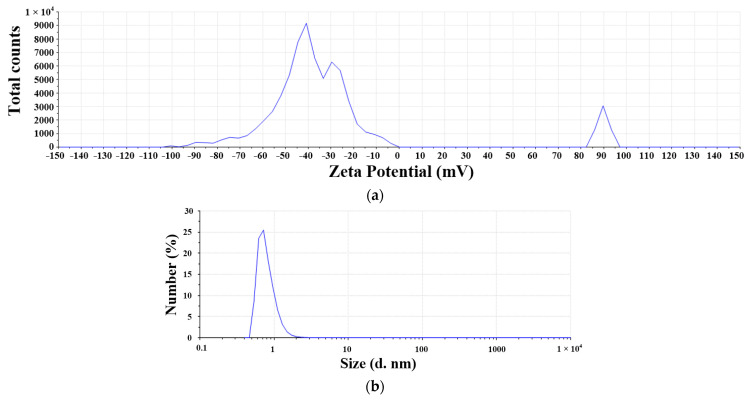
Characteristics of nanosilver colloid with a smaller size: (**a**) zeta potential distribution; (**b**) number PSD.

The micro-EDM was used to prepare the nanosilver colloid for testing solution NO.3. In addition, the zeta potential was −40 mV, as shown in Figure 10a. The size was around 0.5~1 nm, as shown in Figure 10b. Testing solution NO.4 was a 1000 ppm Ag^+^ standard solution.

### 3.2. Microbial Control Cotton Pad Test Results and Discussion

**Figure 11:**
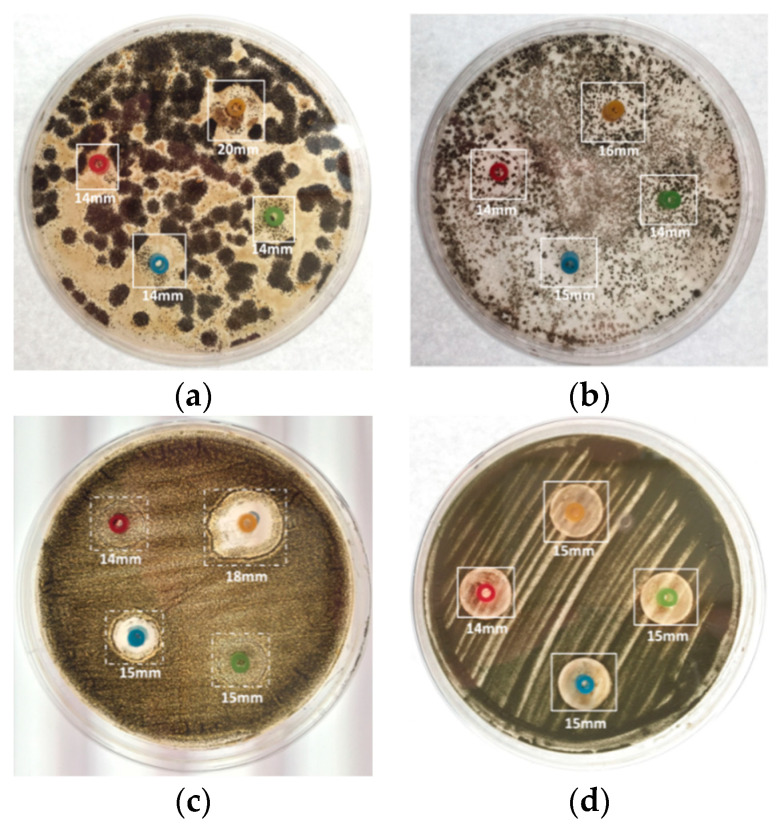
Pictures of inhibition zone test: (**a**) bathroom sample; (**b**) Penicillium; (**c**) Aspergillus Niger; (**d**) Aspergillus Flavus.

**Figure 12:**
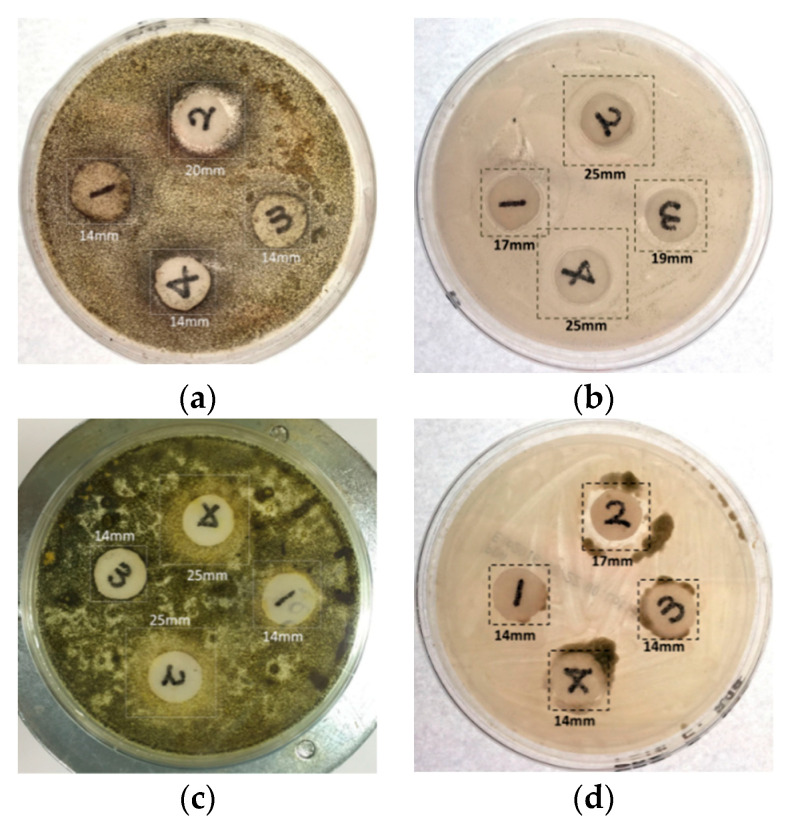
Pictures of cotton pad test: (**a**) bathroom sample; (**b**) Penicillium; (**c**) Aspergillus Niger; (**d**) Aspergillus Flavus.

The experimental results of the inhibition zone test (metal ring) are shown in Figure 11. The experimental results of the inhibition zone test (cotton pad) are shown in Figure 12.

### 3.3. The Results and Discussion of Inhibition Zone Tests

**Figure 13:**
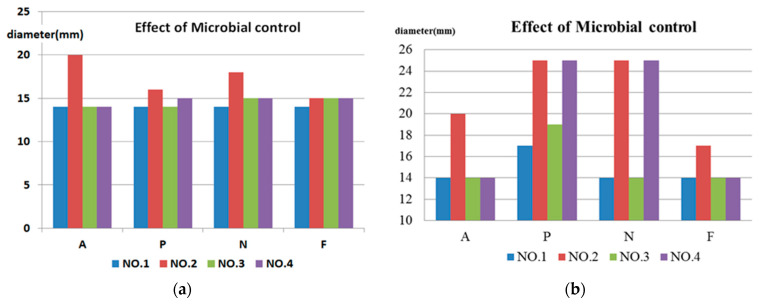
Effects of microbial control: (**a**) metal ring and (**b**) cotton pad.

The results of the inhibition zone tests are described as follows. In addition, the results are shown in Table 8 and Table 9 and Figure 13 [46,47].

1. In the inhibition zone test, the metal ring was placed on the culture medium, and the test solution was then injected into the metal ring [48]. The test solution was slowly absorbed by the culture medium before the metal ring was removed. If the metal ring is removed before the test solution is dried up, the flow of the test solution may influence the experimental results.
2. The microbial control cotton pad test was an improvement proposed for inhibition zone testing [49,50]. Compared to the experiment with the metal ring, in which the test solution needs to be injected in the middle of the ring, the cotton pad holds water, so the test solution will not flow on the culture medium, and the waiting time for drying during the experiment can be shortened.
3. The microbial control cotton pad test results were relatively objective and observable. It was observed that the NO.2 and NO.4 test solutions had the best microbial control effects.
4. The inhibition zone test was implemented for four kinds of fungi in the NO.1~NO.3 nanosilver colloids with different concentrations and characteristics and the NO.4 Ag^+^ standard solution. It was observed that the NO.2 32 ppm nanosilver colloid had the best microbial control effect, then the NO.4 Ag^+^ standard solution at the same concentration. It may be that, because the silver nanoparticles release Ag^+^ continuously at the same Ag^+^ concentration, the microbial control effect is better than the standard Ag^+^ solution. While the concentration of the NO.1 and NO.3 nanosilver colloids was about 5ppm, NO.3 had the smallest silver nanoparticle, meaning a larger contact surface area and the highest zeta potential; thus, its microbial control effectiveness was better than NO.4.

**Figure 14:**
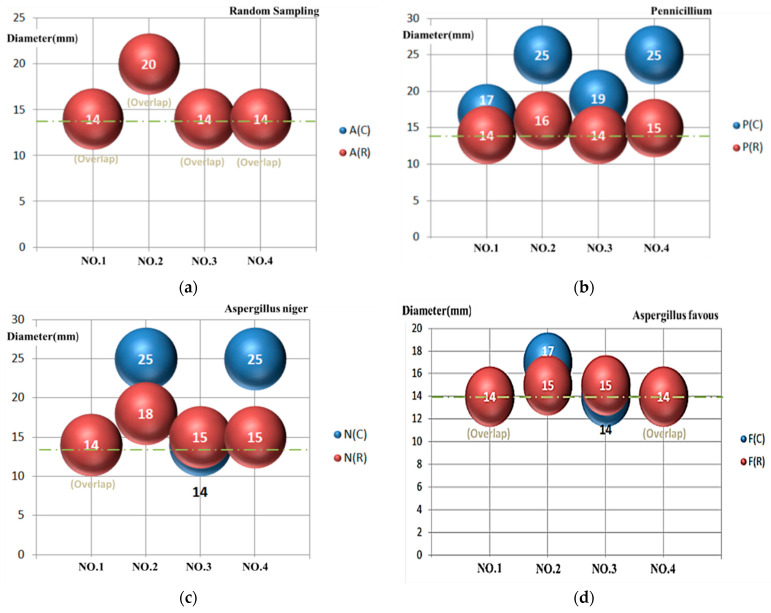
Comparison of microbial control tests: (**a**) bathroom sample; (**b**) Penicillium; (**c**) Aspergillus niger; (**d**) Aspergillus Flavus.

Comparing the two inhibition zone tests, the metal ring limited the expansion of the inhibition zone when the fungus grew, so it is difficult to observe the actual microbial control effect. Figure 14 shows the experimental results of using the metal ring and the cotton pad. The figure “(C)” represents the use of a cotton pad, and “(R)” represents the use of a metal ring. In the NO.1 test liquid, the effect on all four samples was the same in the metal ring experiment, all inhibited to 14mm. In addition, in the cotton pad experiment, the nanosilver had more effect on Penicillium at 17 mm. The NO.2 test liquid had more effect on the bathroom samples at 20 mm but had only a 15 mm inhibited effect on flavus. In the cotton pad experiment, the inhibited effect was more effective in Penicillium and Aspergillus Niger, both with inhibiting effects at 25 mm. The NO.3 and NO.4 test liquids had little difference in effect on the four samples and were all about 14–15 mm in the metal ring experiment. In the cotton pad experiment, NO.3 had more effect on Penicillium at 19 mm, while the effect on the others was 14 mm; and NO.4 had an effect of 25 mm on Penicillium and Aspergillus Niger, and the effect on the others (bathroom and Aspergillus flavus samples) was only 14 mm. The order of the microbial test effect is Penicillium (P) > Aspergillus niger (N) > bathroom sample (A) > Aspergillus flavus (F).

## 4. Conclusions

There are two ways to fabricate nanoparticles: one is physical (the “bottom-up”), and the other is a chemical (the “top-down”) technique. The chemical method uses chemical agents, which is not environmentally friendly. In addition, other physical methods consume energy/time and cannot instantly monitor the process. This study developed a real-time monitoring system for the Electrical Spark Discharge Method (ESDM) using a micro-Electrical Discharge Machine. This study has two limitations. One is that the ESDM is a physical method and, unlike the chemical way, cannot obtain the precise size and concentration of the nanoparticle. The other is that the inhibition of Niger cannot obtain a precise concentration but a relative one. However, all the experiments in this study were conducted with a standardized process in order to reduce the errors made by the human hand. With this system, the micro-EDM can obtain the successful discharge times, electrode energy consumption, and discharge success rate. The conclusions of this study are as follows:

1. The user may adjust the successful discharge times, electrode energy consumption, and discharge success rate at any time to obtain the optimal parameters to fabricate the nanosilver colloid using the Electrical Spark Discharge Method.
2. In the inhibition zone experiment, this study proposes use of the microbial control cotton pad. The microbial control cotton pad is characterized by its low cost, quick experimentation, and easy observation. It is unlikely to fail, and there is no metal ring; thus, the presentation of the inhibition zone will match the actual result better.
3. In the NO.1 liquid, the effects were inhibited at 14 mm in all four samples in the metal ring experiment and at 17 mm in Penicillium in the cotton pad experiment. In the metal ring experiment, the NO.2 liquid had an effect at 20 mm in the bathroom samples and a 15 mm inhibited effect on flavus. In the cotton pad experiment, the inhibited effect was more effective in Penicillium and Aspergillus Niger, both with an inhibited effect at 25 mm. The NO.3 and NO.4 liquids had a14–15 mm effect on all samples in the metal ring experiment. In the cotton pad experiment, NO.3 had an effect on Penicillium at 19 mm, while its effect on the others was only 14 mm. NO.4 had an effect of 25 mm on Penicillium and Aspergillus Niger and only 14mm on the bathroom and Aspergillus flavus samples.
4. The inhibition zone experiment found that the Ag^+^ and nanosilver colloids have a better microbial control effect on penicillium and Aspergillus niger and a worse effect on Aspergillus flavus.
5. Although this study has verified that a nanosilver colloid made using an EDM has microbial control effects, it is only a small step. The issue of nanomaterials is a big subject in terms of antibacterial, biomedical, and environmental aspects. All of the research topics are only just beginning. It is worth investing in more research to benefit more people in the near future.

## Figures and Tables

**Table 1:** Experimental design and configuration.

Strain		Culture Medium
Name	Code	
Bathroom sample	A	MEA
Penicillium	P	MEA
Aspergillus niger	N	MEA
Aspergillus flavus	F	PDA

**Table 2:** Parameter settings of the micro-EDM.

Voltage & Current	V = 100 V, I = 4.2 A	SENS.	Kp = 0.75, Ki = 0.045, Kd = 0.035
T_on_-T_off_	10–10	Z-Axis	off
Capacitor	off	Machining	off
Servo	---	HV	off

**Table 3:** Materials and testing conditions of the micro-EDM.

Diameter of Ag	Anode: 1 mm; Cathode: 2 mm	Beaker	200 mL
Processing time	10 min	Filter paper	Advantec
Dielectric fluid	DW	ATM	1 atm

**Table 4:** Weight loss calculations of the micro-EDM.

Weight		Before W_0_ (mg)	After W_1_ (mg)	W_0_–W_1_ (mg)
Items				
E_1_(anode)		5081.25	5080.96	0.29
E_2_(cathode)		3186.96	3186.87	0.09
E_1_ + E_2_		8268.21	8267.83	0.38
Temperature(°C)		25 °C	28 °C	-----

**Table 5:** Parameter settings of the EDM.

Voltage & Current	V = 140 V, I = 11.8 A	SENS	1/2
T_on_–T_off_	50–50	Z-Axis	off
Capacitor	off	Machining	off
Servo	1/2	HV	off

**Table 6:** Materials and testing conditions of the EDM.

Diameter of Ag	Anode: 1 mm; Cathode: 2 mm	Beaker	250 mL
Processing time	40 min	Filter paper	Advantec
Dielectric fluid	DW	ATM	1 atm

**Table 7:** Weight loss calculations of the EDM.

Weight		Before W_0_ (mg)	After W_1_ (mg)	W_0_–W_1_ (mg)
Items				
E_1_(anode)		640.70	518.49	122.21
E_2_(cathode)		5775.78	5773.56	2.22
E_1_ + E_2_		6416.48	6292.05	124.43
Temperature(°C)		21 °C	32.6 °C	-----

**Table 8:** Results of microbial test (metal ring), diameter (mm).

Test Liquids		NO.1	NO.2	NO.3	NO.4
Samples					
Bathroom (A)		14	20	14	14
Penicillium (P)		14	16	14	15
Aspergillus niger (N)		14	18	15	15
Aspergillus flavus (F)		14	15	15	15

**Table 9:** Results of microbial test (cotton pad), diameter (mm).

Test Solution		NO.1	NO.2	NO.3	NO.4
Samples					
Bathroom (A)		14	20	14	14
Penicillium (P)		17	25	19	25
Aspergillus Niger (N)		14	25	14	25
Aspergillus Flavus (F)		14	17	14	14

## Data Availability

Not applicable.

## Abbreviations

Electrical Discharge Machine (EDM)	Direct Current (DC)
Micro-Electrical Discharge Machine (micro-EDM)	Potato Dextrose Agar (PDA)
Electrical Spark Discharge Method (ESDM)	Bioresource Collection and Research Center (BCRC)
Deionized Water (DW)	Malt Extract Agar (MEA)
